# Electrostatic Design of Polar Metal–Organic Framework Thin Films

**DOI:** 10.3390/nano10122420

**Published:** 2020-12-03

**Authors:** Giulia Nascimbeni, Christof Wöll, Egbert Zojer

**Affiliations:** 1 Institute of Solid State Physics, NAWI Graz, Graz University of Technology, Petersgasse 16, 8010 Graz, Austria; nascimbenigiulia@gmail.com; 2 Institute of Functional Interfaces (IFG), Karlsruhe Institute of Technology (KIT), Hermann-von-Helmholtz Platz-1, 76344 Eggenstein-Leopoldshafen, Germany; christof.woell@kit.edu

**Keywords:** metal–organic frameworks, electronic structure, electrostatic design, density functional theory, work-function change, polar MOFs, bonding asymmetry

## Abstract

In recent years, optical and electronic properties of metal–organic frameworks (MOFs) have increasingly shifted into the focus of interest of the scientific community. Here, we discuss a strategy for conveniently tuning these properties through electrostatic design. More specifically, based on quantum-mechanical simulations, we suggest an approach for creating a gradient of the electrostatic potential within a MOF thin film, exploiting collective electrostatic effects. With a suitable orientation of polar apical linkers, the resulting non-centrosymmetric packing results in an energy staircase of the frontier electronic states reminiscent of the situation in a pin-photodiode. The observed one dimensional gradient of the electrostatic potential causes a closure of the global energy gap and also shifts core-level energies by an amount equaling the size of the original band gap. The realization of such assemblies could be based on so-called pillared layer MOFs fabricated in an oriented fashion on a solid substrate employing layer by layer growth techniques. In this context, the simulations provide guidelines regarding the design of the polar apical linker molecules that would allow the realization of MOF thin films with the (vast majority of the) molecular dipole moments pointing in the same direction.

## 1. Introduction

Metal–organic frameworks (MOFs) consist of metal–oxo nodes connected by di- or higher-topic organic linkers. They form crystalline, highly regular, and porous structures [1]. The high variability of possible node structures and linkers has resulted in the synthesis of tens of thousands of different MOF structures with hugely differing properties [2]. Relying on their highly porous structure, MOFs have traditionally been employed in areas such as catalysis, [3,4,5] gas storage, [6,7,8] and gas separation [9,10]. More recently, their optical and electronic properties have also gained considerable interest [11,12,13,14,15], resulting in applications such as sensing [12,16] and light harvesting [17,18,19]. On more fundamental grounds, in recent years, the dynamics of charge carriers [13,20,21,22,23,24] and excitons [25,26,27,28] in MOFs have attracted considerable interest.

For many of the envisioned (opto)electronic applications of MOFs, strategies for designing their electronic properties would be of distinct relevance. A highly promising approach for locally manipulating the electronic structure is electrostatic design. It relies on the fabrication of structures containing periodic arrangements of polar entities [29,30,31]. The superposition of the fields of the periodically arranged dipoles results in so-called collective (also termed cooperative) electrostatic effects, which are commonly observed at organic–inorganic hybrid interfaces [32,33,34,35,36]. They originate from the fact that the extended two dimensional layer of dipoles rigidly shifts the electrostatic energy of electrons between the regions above and below the layers with the magnitude of the effect being proportional to the dipole density [32,33,34,35,36]. 

**Figure 1:**
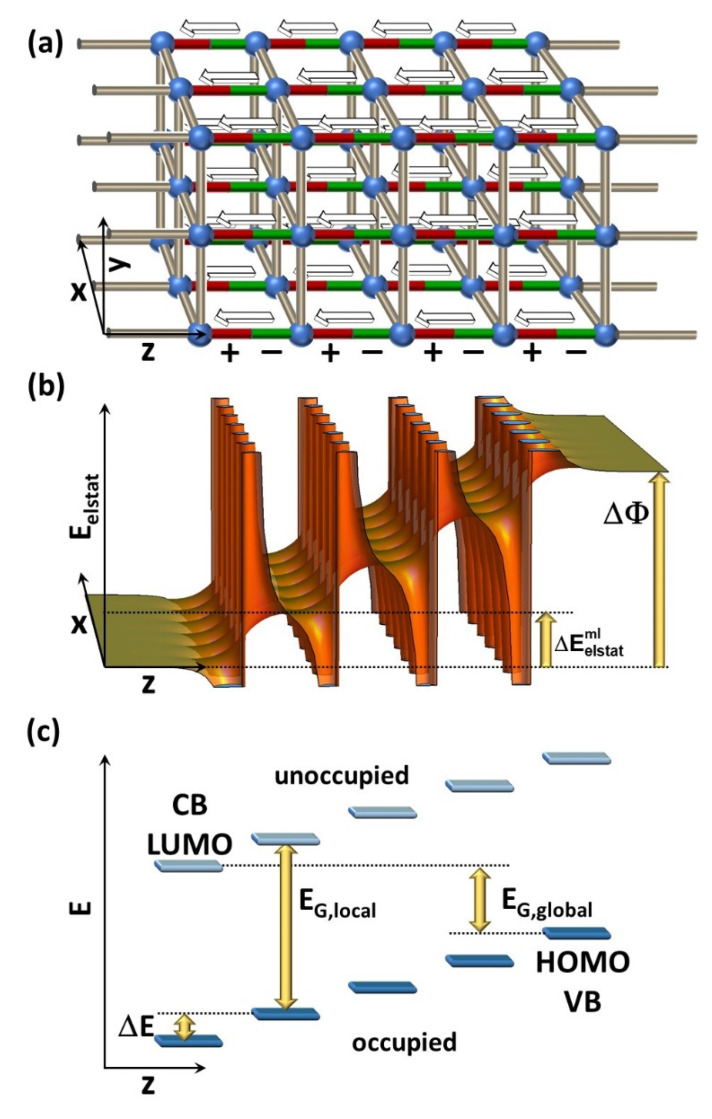
Design and properties of a polar metal–organic framework. Panel (**a**) shows the possible structure of a polar metal–organic framework (MOF) in which the nodes (blue spheres) are connected by regular (apolar) linkers in x- and y-directions (grey cylinders) and by polar linkers in z-direction (red/green cylinders). The direction of the dipole moments is indicated by arrows. (**b**) Change in the electrostatic energy of an electron, E_elstat_, induced by a series of four dipole sheets, which are infinitely extended in x- and y-directions. The dipoles in the sheets point in z-direction and they are arranged on a square lattice with periodicity, D. The distance between the dipole sheets also amounts to D and the plotting range to 6·D in x- and z-directions. The actual value of D is inconsequential for the shape of the plot, as is the case also for the magnitude of the individual dipole moments. (**c**) Impact of the dipole layers on the frontier electronic states, which are assumed to be localized above, below, and between the dipole layers, are shown in panel (**a**). These could, for example, be the electronic states in the MOF localized on layers formed by nodes and apolar linkers, as described in the main text. The various physical quantities contained in the plot are defined in the main text.

Consequently, arranging dipoles in multiple, consecutive layers into an asymmetric structure, as depicted in Figure 1a, results in an energy staircase. This is schematically shown in Figure 1b,c for a model thin film containing four layers of point dipoles. Here, Figure 1b displays the position dependence of the electrostatic energy of an electron, E_elstat_, as derived from the superposition of the electric fields of the four layers of point dipoles (including the divergence of the potential at the locations of the dipoles). Figure 1c highlights the expected impact of the dipole layers on the electronic states of a layer of semiconducting material sandwiched between the polar layers. More specifically, it describes the relative energetic shifts of the frontier electronic states, denoted as valence-band edge (VB) (or highest occupied molecular orbital (HOMO)) and conduction-band edge (CB) (or lowest unoccupied molecular orbital (LUMO)), respectively.

In the current paper, we employ fundamental electrostatic considerations and density-functional theory based band-structure calculations to predict the consequences of the inclusion of polar layers for the electronic properties of MOF thin films. Moreover, we will discuss a possible synthetic approach for realizing such systems. 

## 2. Electronic Structure of a Polar MOF Thin Film 

The fundamental impact of embedded polar layers on the properties of materials and interfaces can be derived from simple electrostatic considerations and has been described in detail, for example, in reference [36]. In the following, we will briefly recapitulate the most relevant aspects and extend the considerations from [36] to systems containing multiple polar layers stacked on top of each other. We will refrain here from explicitly discussing the difference between stacked dipole layers and stacked individual dipoles and the artifacts arising from employing periodic boundary conditions, which has been discussed in an earlier publication [37]. 

The stepwise change in electrostatic energy due to the presence of a single polar layer, $\Delta E_{elstat}^{ml}$, can be derived from the Poisson equation. It is given by:

(1) $$\Delta E_{elstat}^{ml}\;=\;-\frac{q_{e}\mu }{\varepsilon_{0}A}\approx \Delta E$$

Here, *q_e_* is the charge of an electron, *ε_0_* is the vacuum permittivity, *µ* is the component of each individual dipole moment orientated perpendicular to the layer, and *A* is the area per dipole. Provided that the polar layers do not interfere with the intrinsic electronic properties of the semiconducting material between the layers, the frontier levels between successive semiconducting regions (e.g., layers formed by nodes and apolar linkers) are shifted by an energy, Δ*E*, which amounts to $\Delta E\;=\;\Delta E_{elstat}^{ml}$ (see Figure 1c). In this situation, the local band gap within each region, *E_G,local_*, remains unaffected by the polar layers [37]. Conversely, the global energy gap as the energetic difference between the highest occupied state and the lowest unoccupied state in the entire sample decreases [37]. From Figure 1c, it can be inferred that this decrease amounts to:

(2) $$E_{G,global}\;=\;E_{G,local}-n\times \Delta E$$

In this context, it has to be mentioned that in thermodynamic equilibrium the global gap has to remain ≥ 0 eV. Thus, when *n* becomes ≥ *E_G,local_*/Δ*E*, electron transfer from the rightmost to the leftmost semiconducting region will occur in conjunction with a polarization of the material in between [37,38]. Alternative scenarios to establish thermodynamic equilibrium that have been observed for oxidic surfaces would be atomic rearrangements at the surface and adsorption/desorption processes that compensate for the dipole across the slab [39,40]. In the absence of thermodynamic equilibrium, the electrostatically triggered energy shifts can exceed the band gap. Indeed, for stacked polar molecules on surfaces, energy shifts of up to 28 eV have been measured [41]. 

From a practical point of view, one could envision applying the energy staircase of the electronic levels in Figure 1c to guide the flow of charge carriers, to separate electrons and holes, or to dissociate excitons [30]. In this sense, the energetic staircase is reminiscent of the band diagram of a *pin*-junction typically used in photodetectors and solar cells [42]. There, the linear position dependence of the band edges is not related to collective electrostatic effects. Rather, it originates from the field generated by uncompensated ionized dopants in the depletion regions of the p- and n-doped semiconductors that also extend into the intrinsic region of the junction. Still, the resulting driving force for separating charges is similar to the situation encountered here. The semiconducting elements necessary for (opto)electronic applications, which exploit the energy gradient, could be directly built into the MOF network [12,13]. Considering the highly porous nature of the MOF structures, one could, however, also think of first building the polar structure and then infiltrating it with (semi)conducting entities [13,14,43].

In passing, we note that the shift in electrostatic energy between successive semiconducting regions will not only affect the frontier levels but will also shift core-level binding energies,$\;BE_{X}^{n}$. When referencing them, e.g., to the Fermi level of a (metallic) substrate (vide infra), one should again observe a shift with the number of polar layers, *n*, separating the probed atoms from the substrate. This yields the following expectation of the position dependence of $BE_{X}^{n}$:

(3) $$BE_{X}^{n}\;=\;BE_{X}^{0}-n\times \Delta E$$

X here denotes the specific core level that is investigated (in the following discussion, the Zn_2s_ core level). Equation (3) suggests that, relying on the highly localized initial states, X-ray photoelectron spectroscopy can be used as an experimental tool for mapping the electrostatic shifts discussed here [36,44,45]. Finally, growing the above series of polar layers on a metallic substrate (see below) will change the work function of the substrate, with the net work-function change, ΔΦ, amounting to (see Figure 1):

(4) $$\Delta \Phi \;=\;n\times \Delta E_{elstat}^{ml}$$

## 3. Materials and Methods

### 3.1. Computational Methodology

To quantitatively model the impact of polar layers on the electronic structure of MOFs, we performed quantum-mechanical simulations in the framework of density-functional theory, which has proven to be a powerful tool for understanding metal–organic frameworks [46]. For our studies, we used the FHI-aims code [47,48,49,50]. For many simulations, periodic boundary conditions in conjunction with the repeated slab approach were applied. Periodic replicas of the slab were quantum-mechanically decoupled in z-direction by a 20 Å wide vacuum region. To also decouple the slabs electrostatically, a self-consistently determined dipole layer was included within the vacuum gap [51,52]. Following the results of convergence tests, we chose 6 × 6 × 4 and 4 × 4 × 1 k-points grids for studying the bulk system (vide infra) and finite slabs, respectively. For the calculations, we primarily employed the Perdew–Burke–Ernzerhof (PBE) functional [53,54], which for determining binding energies was coupled to a Tkatchenko–Scheffler type van der Waals correction [55]. The latter had, however, no impact on the dipole moments, which determine the electronic structures of the systems, and also only weakly modified bonding asymmetries, as discussed in the Supplementary Materials. As far as the basis set is concerned, the FHI-aims default light settings, tier 2 [47], were used for every atom. A more detailed description of the used basis sets is again found in the Supplementary Materials. To assess the impact of the basis set superposition error when calculating bonding energies, we performed tests for the 3,5-difluoro-4,4′-bypiridine-based system, increasing the basis set size and employing a counterpoise correction. In both cases, changes in bonding asymmetries were ≤ 1 meV, as shown in the Supplementary Materials. Convergence criteria for the self-consistency cycle were set to the default values for changes in the charge density (10^−5^), the total energy (10^−6^ eV) and the forces (10^−4^ eV·Å^−1^). The geometry optimizations were performed using the trust radius method enhanced version of the Broyden–Fletcher–Shanno–Goldfarb optimization algorithm enhanced by the trust radius method [47], with a tolerance threshold of 10^−2^ eV·Å^−1^. To determine the occupation of the Kohn–Sham eigenstates, a Gaussian broadening function with a width of σ = 0.01 eV was used. For the system with 7 layers of polar linkers, the value had to be increased to 0.02 eV to reach convergence (due to the closing of the global gap). Test calculations on thinner layers with the increased broadening did not result in any appreciable changes of the electronic structure compared to the original 0.01 eV broadening. As the studied system contains Zn atoms, the atomic ZORA approximation (zero-order regular approximation for relativistic effects) was used [56] to account for relativistic effects. Density of state (DOS) and projected density of state (PDOS) plots were obtained using the same Gaussian broadening function with a broadening of 0.01 eV as in the self-consistent field procedure. The band gap (Kohn–Sham gap) as a function of *n* was obtained as the energy difference between the lowest unoccupied and the highest occupied states (for the above-described k-point sampling). Work function differences were derived from the energetic difference between the Fermi level and the vacuum level below and above the slab, respectively. Core-level energies were calculated within the initial-state approach [57,58,59,60] as energies of the Zn_2s_ orbitals in the respective layers.

As generalized gradient functionals such as PBE severely underestimate band gaps, to get somewhat improved values, the electronic structures of selected systems were recalculated using the hybrid Heyd–Scuseria–Ernzerhof (HSE06) exchange correlation functional [61]. These calculations were performed using a screening parameter of omega = 0.11 Bohr^−1^ [62], light settings and a tier 1 basis set with the further addition of the first two radial functions of the tier 2 basis set (a full tier 2 basis would have been computationally too expensive). The use of fewer basis functions for the HSE06 calculations is justified by convergence tests, which showed that there were no relevant differences between the results obtained with the full and the reduced tier 2 set. Due to the high computational costs of hybrid calculations combined with periodic boundary conditions, with HSE06 only single point calculations on the PBE optimized structures were performed. Plots of the electrostatic energy were produced using XCrySDen [63] and three dimensional geometries were plotted using OVITO [64].

As far as the geometries of the investigated systems are concerned, an optimization of the bulk geometry of the di-zinc secondary building units (SBUs) connected in the x,y-plane by terephthalic units and in the z direction by 3,5-difluoro-4,4′-bypiridine molecules yielded a tetragonal unit cell. This prompted us to also keep mutually orthogonal edges for all derived structures. Starting from that bulk structure, as a first step, the H atoms in positions 3 and 5 in the bipyridine system were replaced by F atoms. The resulting structure was relaxed, simultaneously optimizing atomic positions and unit cell dimensions. Based on the optimized bulk structure, a monolayer slab was constructed, consisting of two layers of SBUs linked by 1,4-benzenedicarboxylate (BDC) units and separated by a monolayer of 3,5-difluoro-4,4′-bypiridine molecules as apical linkers. The terminal Zn atoms were saturated with pyridines (see Figure 1c). Atomic positions and unit-cell dimensions in the x and y directions were optimized. As in this process the lengths of the unit-cell vectors did not change compared to the bulk, they were kept fixed for all further calculations. Systems with larger numbers of polar layers were constructed in an analogous fashion, where the atomic positions were fully optimized for systems containing up to 3 polar layers (*n* = 3). As these optimizations yielded only very minor relaxations, thicker slabs were generated by replicating the 3,5-difluoro-4,4′-bipyridine (m2F-BP) layer and one of the central layers of BDC-linked Zn-paddlewheels without further geometry optimizations. 

### 3.2. Suggesting a Strategy for Realizing a Polar MOF

Conceptually, for realizing MOFs comprising a finite number of polar layers (as depicted in Figure 1a), three main criteria have to be fulfilled: (i) polar linkers have to be incorporated in an oriented fashion, aligned only along one spatial direction, (ii) the (vast) majority of the linkers must be aligned with their dipoles pointing in the same direction, yielding a non-centrosymmetric thin film, and (iii) one must be able to grow a finite, well defined number of layers comprising polar linkers, potentially sandwiched between layers in which the linkers are apolar.

Ideally suited for achieving the criteria, especially (i) and (iii), are layer-by-layer growth techniques [65,66,67,68] applied to surface-mounted MOFs (SURMOFs) [66,68]. Here, the substrate is typically functionalized with suitable anchoring groups [69], then exposed to a metal source (e.g., Zn-acetate), rinsed, exposed to one type of linker, rinsed, potentially exposed to another type of linker, rinsed etc. For growing polar MOFs, one needs nodes that bond differently in different directions; then, one has to introduce polar linkers in the step that triggers the growth of the MOF in the direction perpendicular to the substrate (the z-direction). Following this procedure, heterolayers [70] can also be produced, where in the present context varying between polar and apolar linkers along the z-direction (perpendicular to the substrate) or varying the dipole densities would be particularly interesting. 

**Figure 2:**
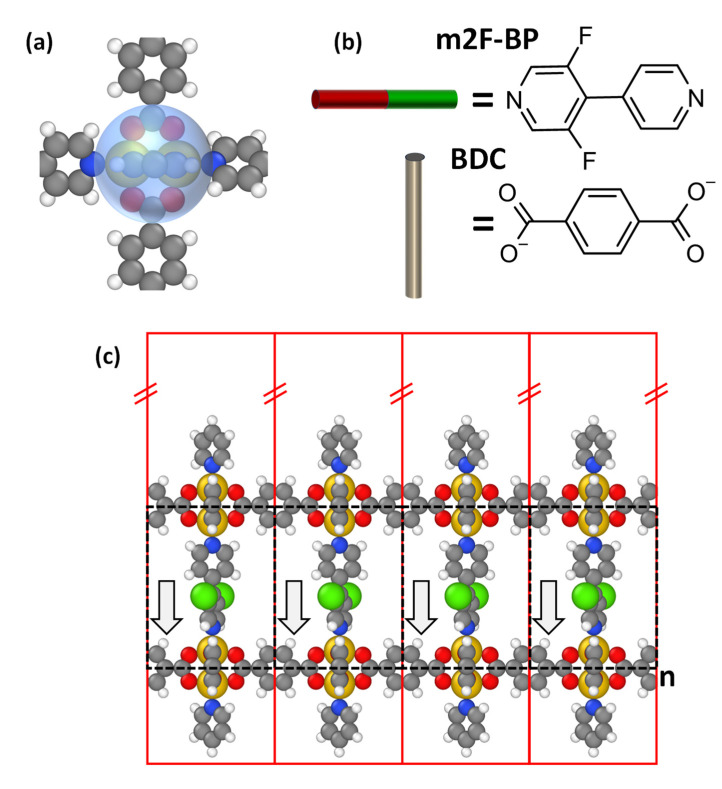
(**a**) Chemical structure and bonding geometry of the Zn-paddlewheel node. The node consists of two Zn atoms connected by four carboxylic acid residues. Parts of the 1,4-benzenedicarboxylate (BDC) linkers and the apical pyridine groups are also shown. The semitransparent blue sphere in the above plot corresponds to the blue sphere in the schematic structure shown in Figure 1a. (**b**) Chemical structures of the linkers: 1,4-benzenedicarboxylate (BDC) corresponds to the grey cylinders from Figure 1a and 3,5-difluoro-4,4′-bipyridine (with F in meta position, m2F-BP) corresponds to the red/green cylinders. (**c**) Structure of a polar monolayer consisting of one layer of polar m2F-BP molecules, two layers of BDC-linked Zn-paddlewheels, and pyridine layers at the top and bottom to saturate the paddlewheels. Each red rectangle denotes the unit cell used in the band structure calculations (vide infra); the dashed black rectangles show the structure that is repeated when the number of polar layers in the slab is increased. The direction of the molecular dipoles is indicated by the arrows. Color code: grey: C; white: H; dark blue: N; red: O; yellow: Zn; green: F.

For discussing the concept of electrostatically designing the energy landscape of MOFs, we start from a prototypical layered-pillar SURMOF, built from so-called Zn-paddlewheels connected by 1,4-benzenedicarboxylates (BDC) (see Figure 2a,b). The resulting 2D planar structures are then connected, e.g., by pillars in the form of bipyridines. For realizing polar structures, the (symmetric) bipyridines need to be replaced by (asymmetric) polar analogues. One of the simplest possibilities is to replace the H atoms in positions 3 and 5 in one of the pyridines by fluorine atoms (m2F-BP, see Figure 2b).

The major challenge in growing polar, non-centrosymmetric MOFs is to align all (or at least the vast majority) of the linkers so that their dipoles point in the same direction. From an electrostatic point of view, such a structure should be energetically unfavorable. Moreover, even if the dipole–dipole repulsion was weak due to the porous structure of the MOF (vide infra), entropy would favor a random orientation of the dipoles of the apical linkers. A possible strategy for overcoming these challenges would be to induce a large enough bonding asymmetry between the two pyridine docking sites in each linker molecule and the Zn-atoms, a topic that will be discussed in detail in Section 4.2.

For simulating polar MOFs, it is necessary to model systems of finite thickness (as in a bulk calculation, the effect of the dipoles would vanish due to the 3D periodic boundary conditions) [37]. Additionally, from a practical point of view, such polar MOF thin films should be easier to realize than a polar bulk. Thus, as model systems, we chose finite numbers of 2D periodic MOF layers consisting of BDC-linked Zn-paddlewheels, connected along the z-direction by m2F-BP linkers. These MOF thin films were then modeled employing the repeated slab approach, as described in the Methods section. As indicated in Figure 2c, such a slab contains *n* layers of polar m2F-BP molecules and *n* + 1 layers of BDC-linked Zn-paddlewheels. The paddlewheels at the top and bottom of the slab are saturated by pyridine layers. Considering the lateral translational symmetry of the system, the unit cell can be chosen so that each layer contains only one Zn-paddlewheel, two BDC linkers, and one m2F-BP molecule (see Supplementary Materials). In x- and y-directions, this unit cell is periodically repeated to represent the laterally quasi-infinitely extended MOF. 

## 4. Results and Discussion

### 4.1. Electronic Structure of the Polar MOF Thin Films

**Figure 3:**
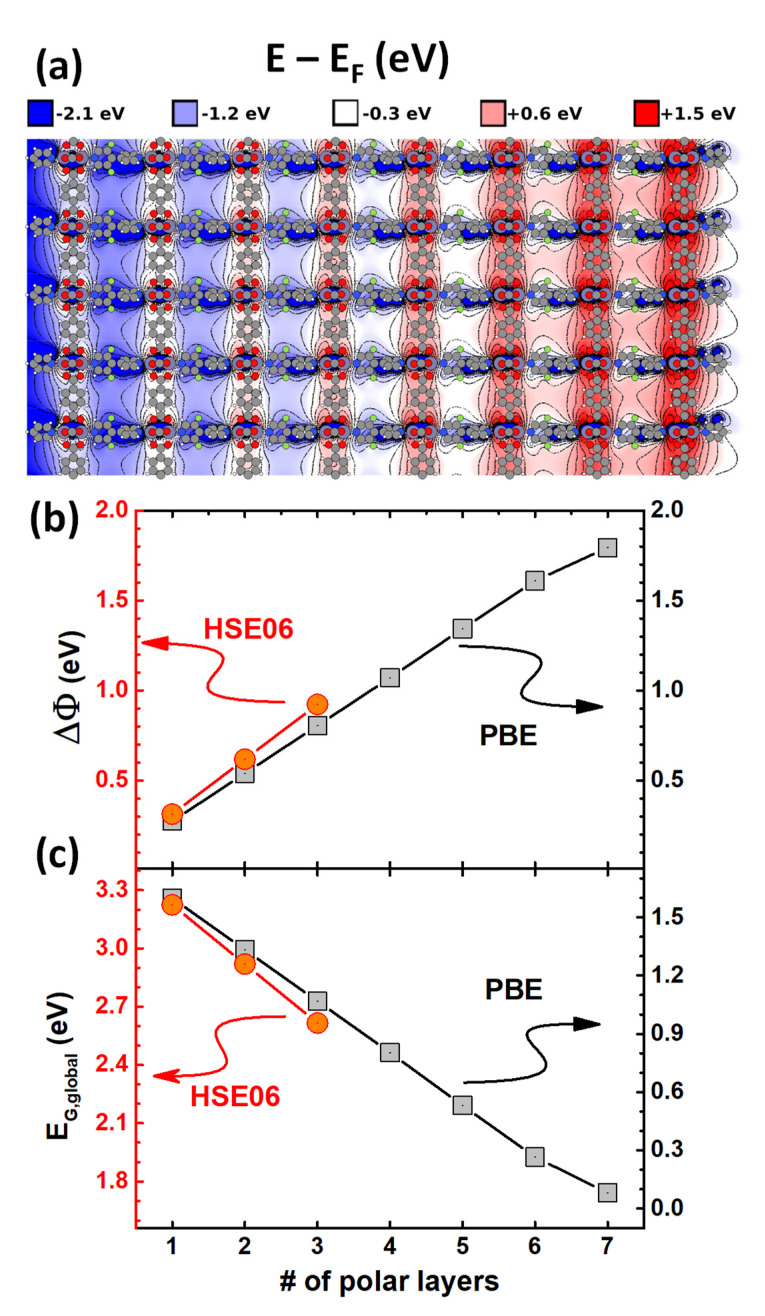
(**a**) Evolution of the density functional theory (DFT)-calculated electrostatic energy of an electron for a MOF thin film containing seven layers of polar, apical m2F-BP linkers. The graph shows the energy in a plane parallel to the x- and z-axes with the y-position chosen halfway between lines of linker molecules; (**b**,**c**) show the evolution of the overall step in electrostatic energy (the work-function change) and the global energy gap as a function of the number of layers of polar, apical m2F-BP linkers. The Perdew–Burke–Ernzerhof (PBE) results are plotted in black (right axes) and the Heyd–Scuseria–Ernzerhof (HSE06) results in red (left axes).

To demonstrate the key features of the electronic properties of polar MOF thin films, we first analyzed the electrostatic energy landscape of a model system containing seven layers of properly aligned m2F-BP linkers. The corresponding plot of the electrostatic energy in the x-z plane halfway between neighboring m2F-BP molecules is shown in Figure 3a for a thin film containing seven layers of polar linkers (*n* = 7). The energy staircase is clearly visible and is particularly well resolved when comparing the regions of the BDC-linked Zn-paddlewheels layers. A quantitative analysis reveals that the energy shift between adjacent layers amounts to ~0.26 eV per layer. An alternative way of visualizing the energy staircase would be to plot the dependence of the electrostatic energy averaged over planes perpendicular to the direction of the dipoles. The corresponding plots are shown in the Supplementary Materials, again for the *n* = 7 situation and for a system consisting of a MOF thin film with four layers of polar apical linkers sandwiched between MOFs comprising two apolar apical linker layers on each side (i.e., the electrostatically generated “pin” junction mentioned above).

When comparing model systems with varying numbers of polar layers, *n*, the energy steps result in a linear increase in the overall shift in electrostatic energy along the *z*-axis of the polar thin film, ΔΦ, with *n* (see Figure 3b). When such a thin film is grown on a metallic substrate with the z-direction normal to the surface, this results in an equivalent work-function shift as expressed by Equation (4). Concomitantly, Figure 3c shows a linear decrease in the global gap with *n*, as predicted by Equation (2). The observation of such a linear decrease instead of a 1/*n* dependence of the gap (as encountered in conjugated oligomers and polymers) [71] confirms that the gap reduction is triggered by electrostatic effects rather than by an increasing conjugation. In passing, we note that a similar observation has been made when calculating the properties of densely packed monolayers consisting of oligopyrimidine molecules [37]. 

For simulations employing the PBE functional [53,54], the above trend prevails up to six polar layers (*n* = 6). For the seventh layer, the gap closes and therefore the increase in ΔΦ between *n* = 6 and *n* = 7 amounts to only 0.18 eV. As a consequence, the shift per layer is somewhat reduced from ~0.27 eV for *n* ≤ 6 to less than 0.26 eV for *n* = 7. Note that generalized gradient functionals such as PBE substantially underestimate the band gap. As a result, the number of layers at which the gap closure occurs is too small in the PBE calculations. In order to improve the situation, we also performed calculations with the hybrid functional HSE06 [61], for which the underestimation of the gap is less serious. Unfortunately, with HSE06 in conjunction with periodic boundary conditions, one encounters sharply increased computational costs. As a result, we only considered MOF thin films containing up to three polar layers. Nevertheless, extrapolating the HSE data from Figure 3c suggests that the gap closure for the more realistic hybrid functionals will take place at around *n* = 12. In passing we note that the somewhat steeper increase (decrease) in ΔΦ (E_G,global_) in the HSE06 calculations compared to the PBE results is the consequence of a larger dipole moment of the m2F-BP molecule for the former functional.

**Figure 4:**
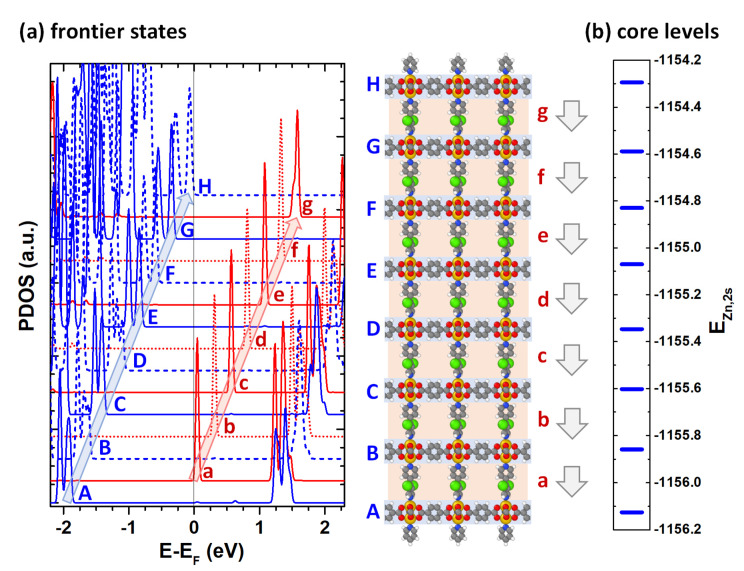
(**a**) DFT-calculated density of states projected onto the m2F-BP layers (red curves, denoted by small letters) and onto BDC-linked Zn-paddlewheels layers (blue curves, denoted by capital letters) for the *n* = 7 thin film. The naming of the layers is shown in the central panel. (**b**) Zn_2s_ core level energies for successive BDC-linked Zn-paddlewheel layers (the plot contains the average value for the two Zn-atoms in one node; for additional details see main text).

In order to analyze local gaps and the energetic shifts between consecutive layers, we calculated the densities of electronic states projected onto the respective sub-systems [37]. These are shown for the valence region of the PBE calculations for the *n* = 7 system in Figure 4a (with a plot covering a wider energy range contained in the Supplementary Materials). Here, projections onto layers of BDC-linked Zn-paddlewheels are denoted by uppercase letters and projections onto m2F-BP layers are denoted by lowercase letters. The calculated local densities of states of adjacent equivalent layers have the same overall shape and are rigidly shifted by ~0.26 eV relative to each other. As discussed in Section 2, this can be attributed to the shifts in electrostatic energies (see Figure 1c). When comparing the chemically different BDC-linked Zn-paddlewheels layers and the layers consisting of m2F-BP linkers (e.g., layers A and a in Figure 4) one observes a difference in the onsets of the occupied (unoccupied) DOSs, which amounts to 1.75 eV (1.19 eV). Overall, despite the decreasing global gap discussed above, the local gaps in the different sub-systems remain constant throughout the entire thin films. It amounts to 3.04 eV in the BDC-linked Zn-paddlewheels layers and to 3.64 eV in the m2F-BP layers. The HOMO (VB edge) is localized on the topmost BDC-linked Zn-paddlewheel layer (layer H), while in the PBE calculations, the LUMO (CB edge) is calculated to be in the lowest m2F-BP layer (layer a). 

As a last aspect, we assess the impact of the polar layers on the core-level binding energies, as via x-ray photoelectron spectroscopy (XPS) these should serve as an experimentally accessible probe for the shifts in electrostatic energy [72]. To this aim, Figure 4b show the binding energies of the Zn_2s_ core levels for the seven layer system calculated within the initial state approach [57,58,59,60]. Core-level shifts are typically well reproduced by this method [73,74,75,76,77], even though the absolute core-level energies need to be rigidly sifted to be compared to experiments (as in the simulations they are associated with the energies of Kohn–Sham orbitals and screening is neglected). The calculated overall shift between the Zn-atoms in the top and bottom layers amounts to as much as 1.83 eV, which (again) corresponds to ~0.26 eV per layer. This implies that the effect described here should be easily identified in XP spectra. Moreover, provided that for a specific system the number of polar layers was known (e.g., from the SURMOF production process), the actually observed XPS shift would yield information on the degree of alignment of the dipoles of the apical linkers. 

The above results show that the expectations from the purely electrostatic considerations in Section 2 are fully met by the results of the quantum-mechanical calculations. One of the reasons why the simple models from Section 2 work out so well here is that the BDC and m2F-BP linkers are quantum-mechanically decoupled from each other by the nodes, i.e., the different sub-systems do not interact strongly with each other. Thus, the electronic states can be efficiently shifted relative to each other by the variations in the electrostatic potential. After discussing the expected electronic structures of polar MOF thin films, we next address how simulations can help address the challenges one will encounter in assembling ordered, all-parallel, non-centrosymmetric arrangements of the polar linkers.

### 4.2. Aligning the Polar Linkers during MOF Growth—Bonding Asymmetry

A common strategy for growing polar layers at interfaces is the use of anchoring groups, such as thiolates on Au. In such systems, each molecule typically contains a single thiol group [78,79,80,81]. Its bonding to the substrate aligns the molecules, provided that the bonding strength is large enough to overcome the dipole–dipole repulsion effects between adjacent moieties. Compared to polar, tightly packed, thiolate-bonded monolayers on surfaces, the dipole density is strongly reduced in highly porous MOFs. As a result, dipole–dipole repulsion effects are substantially reduced (vide infra) and thus should loosen the requirements for the docking groups. Still, they need to bond strongly enough to stabilize the MOF structure and weakly enough to break and reform in the self-assembly processes during MOF growth. The main challenge is, however, that in order to grow 3D structures, the linkers need to contain two docking functionalities. A possible strategy for aligning the dipoles could be layer-by-layer growth of the MOF (vide supra) in combination with asymmetric linkers containing two distinctly different docking groups, one of which binds to the nodes with significantly higher bonding strength than the other. 

To discuss this on a more quantitative level, we first calculated bonding energies and their asymmetries for several chemically related linkers with regard to the pillared layer MOFs discussed above. 

**Figure 5:**
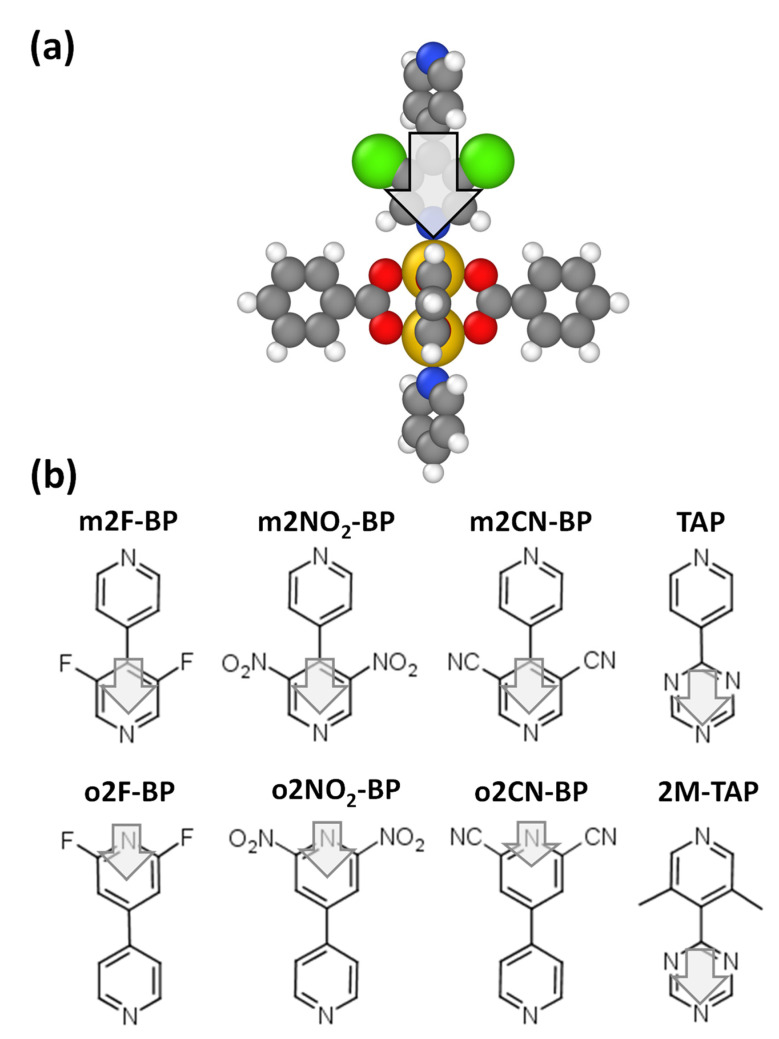
(**a**) Structure of a saturated Zn-paddlewheel monomer bonded to a polar apical linker (here m2F-BP) in the “down” orientation (gray: C; white: H; blue: N; red: O; yellow: Zn). (**b**) Chemical structures of the polar apical linkers considered in the present study: 3,5-difluoro-4,4′-bipyridine (with F in meta position, m2F-BP), 2,6-difluoro-4,4′-bipyridine (with F in ortho position, o2F-BP), 3,5-dinitro-4,4′-bipyridine linker (m2NO_2_-BP), 2,6-dinitro-4,4′-bipyridine linker (o2NO_2_-BP), 3,5-dicyano-4,4′-bipyridine (m2CN-BP), 2,6-dicyano-4,4′-bipyridine (o2CN-BP), 4-s-triacinylpyridine (TAP), 3,5-dimetil-4-s-triacinylpyridine (inducing a twist, 2M-TAP).

To separate the impact of the bonding from electrostatic interactions between neighboring polar linkers, the bonding energies were first calculated for monomer systems consisting of only a single (saturated) Zn-paddlewheel unit as shown in Figure 5a. In the following discussion, “up” and “down” refer to the orientation of the linker dipole. The systems considered in addition to m2F-BP are shown in Figure 5b and all simulated monomer structures can be found in the Supplementary Materials. All considered linkers are based on bipyridine backbones, so that they can be tested in analogous MOF configurations and could also be used to grow polar MOF heterostructures, employing the growth techniques described in [70].

Table 1 contains the dipole moments of the isolated molecules and of the “up” and “down” monomers. Additionally, the bonding energies and their asymmetries in the above-mentioned monomer systems are listed. Bonding energies, *E_b_*, are defined as the difference between the energies of the system with the linker bonded to the paddlewheel and of the two isolated sub-systems (polar linker and paddlewheel monomer with the linker removed). For the determination of *E_b_*, the geometries of all (sub)systems were optimized disregarding solvent effects.

The data for the isolated linker molecules in Table 1 show that m2F-BP has the smallest dipole moment of all considered apical linkers; the dipole moment of 4-s-triacinylpyridine (TAP) is nearly twice as large and the dipole moments increase further for 2,6-difluoro-4,4′-bipyridine (o2F-BP), 3,5-dimetil-4-s-triacinylpyridine (2M-TAP) and 3,5-dicyano-4,4′-bipyridine (m2CN-BP). Particularly large dipole moments are obtained for –CN and –NO_2_ substituents in 2,6- (i.e., in ortho) positions. Considering the monomer dipoles in down orientation (i.e., the clusters also containing a saturated paddlewheel), one still observes the smallest value of µ_down_ for m2F-BP. The difference between molecular and cluster dipoles, however, varies considerably. This shows that the polarity of the Zn–N bond is significantly impacted by the substitution pattern of the apical linker. A consequence of this is, for example, that periodic monolayers of m2F-BP-linked MOFs have energetic steps per polar monolayer that are rather similar to their o2F-BP-linked counterparts (see Supplementary Materials), despite the significantly different molecular dipoles of the linker molecules. This can be rationalized by the estimates for the overall dipoles of two saturated paddlewheels connected by a polar linker, Δµ, in Table 1. 

As far as the energetic stabilities of the N–Zn bonds are concerned, they are rather similar for all systems with the notable exceptions of E_b,up_ for o2F-BP and o2CN-BP. The latter two are strongly reduced, which we attribute to steric repulsions between the node and linker molecule due to the F atoms or –CN groups in ortho position. As a consequence, of all linkers shown in Figure 5, o2F-BP and o2CN-BP are the only ones with an appreciable asymmetry in the bonding energies, ΔE_b_, i.e., these two ortho-linked systems hold the highest promise for a spontaneous alignment of the polar linker. A possible complication when exploiting the above-mentioned steric effects is, however, that the reduced binding energies for the up configuration might destabilize the entire structure. This becomes apparent for the o2NO_2_-BP molecule attached to a Zn-paddlewheel in up orientation, where we calculated a strongly distorted structure with a broken N–Zn bond (see Supplementary Materials), which would prevent the formation of a 3D MOF. 

A few additional aspects are worth mentioning in the context of the energetic stability of aligned polar monolayers: (i) for the “up” version of the m2F-BP monomer, we also calculated the total energy of the system as a function of the distance between the node and the m2F-BP linker. This yielded a monotonically increasing curve (see Supplementary Materials), indicating that there would be no activation energy barriers impacting the kinetics of bond formation and bond breaking. 

(ii) As mentioned above, due to the reduced dipole density in the porous MOF, one can expect that dipole–dipole interactions within a linker layer should be strongly reduced compared to, e.g., a densely packed self-assembled monolayer on the surface. To quantify the effect, we recalculated the bonding energies for the m2F-BP system for a layer of BDC-linked Zn-paddlewheels saturated on one side with pyridines and on the other side with m2F-BP molecules, comparing the situations for all m2F-BP molecules aligned in “up” and “down” orientations. Additionally, we constructed a layer with m2F-BP molecules aligned in an alternating, checkerboard-type fashion (see Supplementary Materials). From an electrostatic point of view, one would expect the checkerboard system to be clearly the most stable one, but the calculations yield the following order of average bonding energies per molecule: E_b,up_ = 1108 meV > E_b,check_ = 1082 meV > E_b,down_ = 1048 meV. This means that for the monolayer, essentially the same bonding asymmetry is obtained as for the monomer calculations (60 meV vs. 66 meV in Table 1). The checkerboard arrangement of the m2F-BP molecules is more or less halfway between the two other configurations (E_b,check_ is only 4 meV higher than the average of E_b,up_ and E_b,down_). This implies that dipole–dipole repulsion plays a close to negligible role for the possible formation of a polar m2F-BP layer. To rationalize this a priori somewhat unexpected finding, one has to keep in mind that due to the porous nature of the MOF, the lateral distance between m2F-BP molecules is approximately 11 Å; moreover, the involved dipole moments are only moderately large. Indeed, a back of the envelope calculation reveals that the repulsion energy between two parallel dipoles of 0.76 Debye at a distance of 11 Å amounts to only 0.3 meV, which for a periodic arrangement of dipoles becomes 1.2 meV (employing a square Topping model [82]). These particularly small values suggest that even when using more polar apical linkers, dipole–dipole repulsion effects should typically be of only minor relevance.

(iii) Likewise, the interaction energy between dipoles in consecutive m2F-BP layers is negligibly small (see Supplementary Materials). This is again a consequence of large inter-dipole distances in conjunction with the much more rapid drop of the electric field outside a periodic dipole layer compared to an isolated dipole [32]. 

In this context, it should, however, be stressed that even if dipole–dipole repulsion effects do not influence the formation of linker layers with aligned dipole moments, entropy will still favor a random orientation of the dipoles, i.e., one still needs to develop energetic driving forces (such as the above-mentioned bonding asymmetry) for growing polar MOF thin films. It should also be mentioned that the arrangement of (polar) solvent molecules relative to the bonding partners will have an impact on actual bonding energy values, but we do not expect them to impact the general conclusions drawn above. 

In passing, we note that an alternative approach for achieving MOFs with aligned dipole moments could be the use of suitably chosen protecting groups, which ensure that, in a first step, only one of the bonding sites of the apical linker can connect to the Zn-paddlewheels. 

## 5. Conclusions

We propose a strategy for controlling the electronic structure of MOF thin films exploiting collective electrostatic effects. In particular, we show how the electronic states in a pillared layer SURMOF can be shifted between consecutive layers by using bipyridines in which one of the pyridines is substituted with electron withdrawing F-atoms as apical linkers (connecting the BDC-linked Zn-paddlewheels). When achieving an alignment of all apical, asymmetric linkers with the dipole moments arranged in a parallel fashion, the observed energetic shift per layer amounts to ~0.27 eV. The resulting energy staircase of the frontier levels in successive semiconducting regions (e.g., BDC-linked Zn-paddlewheel layers) is reminiscent of the situation in a pin-photodiode, although the origin of the energy gradient is fundamentally different in both cases (field due to ordered 2D dipole layers vs. space charge regions due to uncompensated ionized dopants). The polar linker layers have virtually no impact on the local energy gaps of individual BDC-linked Zn-paddlewheel sheets. The global energy gap (defined as the energetic difference between the highest occupied and lowest unoccupied states in the entire system) drops, however, linearly with the number of polar layers. Eventually, for a sufficiently large number of polar layers, the global gap even vanishes. In addition to the positions of the frontier levels, the energies of the core levels are also electrostatically shifted. Therefore, core-level spectroscopy appears as an ideal tool for probing the success of the alignment of the dipoles of the apical linkers. 

A possible strategy for realizing the alignment would be to grow the MOFs in a layer-by-layer fashion, exploiting different binding energies of the two complexing units at the ends of the linkers. Exploring this approach by calculations for a variety of polar bipyridine derivatives reveals that the bonding asymmetries caused by polar substituents on only one of the rings become appreciable only when combining them with steric repulsion effects, as encountered for 2,6-substituted linkers. Such systems could, indeed, be promising, considering that dipole–dipole repulsion effects between aligned neighboring apical linkers are very small due to the large inter-dipole distances. However, a possible complication in this context is that the reduced binding energies for 2,6-bonded substituents might adversely affect the stability of the resulting MOFs, suggesting that it would be worthwhile to also explore alternative approaches, such as the use of protecting groups. 

As an outlook, it should be noted that the lack of inversion symmetry in polar MOF thin films also makes them highly promising for first-order nonlinear optical (NLO) applications, with polar apical linkers acting as perfectly aligned NLO chromophores [83]. This should result in much larger nonlinearities compared to MOFs with centrosymmetric structures, which exploit second-order nonlinear effects [84]. In fact, the realization of polar MOFs could boost the applicability of MOFs in nonlinear optics and might well help to overcome the challenge of properly aligning first-order NLO chromophores [83]. This would enable the use of MOFs in applications such as second harmonic generation, or electro-optic switching.

## Figures and Tables

**Table 1:** PBE-calculated values of the molecular dipole moments, µ_mol_ (they are obtained for a downward orientation of the dipoles and thus are reported as being negative); monomer-dipole moments for dipoles in the apical linkers in down orientation, µ_down_, (see Figure 5a) and up orientation, µ_up_; estimate for overall dipole of two saturated paddlewheels connected by a polar linker, Δµ (see below); bonding energy between saturated paddlewheel and apical linkers for dipole-down, E_b,down_, and dipole-up, E_b,up_, orientations; and bonding asymmetry, ΔE_b_ = E_b,up_ − E_b,down_; a negative value implies that the up-conformation is more strongly bonded. Δµ is given by Δµ = µ_up_ − µ_down_ − µ_mol_. The expression can be rationalized by the actual system comprising the bond dipoles of linkers bonded in up- and down orientation (the latter with an inverted sign) plus the dipole of the polar molecule. Bond dipole here refers to the change in dipole between the bonded structure and an isolated molecule. The reason why µ_mol_ has to be subtracted rather than added is that already both µ_up_ and µ_down_ contain polar molecules, albeit in different orientations (for further details see Supplementary Materials).

	m2F-BP	o2F-BP	m2NO_2_-BP	o2NO_2_-BP	m2CN-BP	o2CN-BP	TAP	2M-TAP
µ_mol_ (D)	−0.76	−1.86	−1.92	−4.85	−2.24	−5.09	−1.33	−1.95
µ_down_ (D)	−3.57	−4.27	−5.95	−7.75	−6.20	−8.05	−4.35	−5.02
µ_up_ (D)	−1.40	−0.63	−0.49		−0.32	0.67	−0.67	−0.16
Δµ (D)	−1.41	−1.78	−3.54		−3.64	−3.63	−2.36	−2.91
E_b,down_ (meV)	995	1057	943	1019	949	1028	946	955
E_b,up_ (meV)	1061	590	1028		1022	674	1058	943
ΔE_b_ (meV)	**−66**	**467**	**−85**		**−73**	**354**	**−112**	**12**

## Supplementary Materials

The following are available online at https://www.mdpi.com/2079-4991/10/12/2420/s1, Depictions of unit cells and all studied clusters; additional methodological details; assessment of impact of vdW correction and of the basis-set superposition error; additional data on plane-averaged potentials and densities of states; structures of all considered monomer systems; reasoning for the estimate of the dipole per unit cell from the monomer data; comparison of the properties of periodic m2F-BP and o2F-BP monolayer systems; check for an activation-energy barrier; assessment of dipole-dipole interactions within monolayers; assessment of dipole-dipole interactions between neighboring layers.

## References

[1] Furukawa H., Cordova K.E., O’Keeffe M., Yaghi O.M. (2013). The chemistry and applications of metal-organic frameworks. Science.

[2] Moghadam P.Z., Li A., Liu X.-W., Bueno-Perez R., Wang S.-D., Wiggin S.B., Wood P.A., Fairen-Jimenez D. (2020). Targeted classification of metal–organic frameworks in the Cambridge structural database (CSD). Chem. Sci..

[3] Liu J., Chen L., Cui H., Zhang J., Zhang L., Su C.Y. (2014). Applications of metal-organic frameworks in heterogeneous supramolecular catalysis. Chem. Soc. Rev..

[4] Zhu L., Liu X.Q., Jiang H.L., Sun L.B. (2017). Metal-Organic Frameworks for Heterogeneous Basic Catalysis. Chem. Rev..

[5] Pascanu V., González Miera G., Inge A.K., Martín-Matute B. (2019). Metal-Organic Frameworks as Catalysts for Organic Synthesis: A Critical Perspective. J. Am. Chem. Soc..

[6] Eddaoudi M., Kim J., Rosi N., Vodak D., Wachter J., O’Keeffe M., Yaghi O.M. (2002). Systematic design of pore size and functionality in isoreticular MOFs and their application in methane storage. Science.

[7] Rowsell J.L.C., Yaghi O.M. (2006). Effects of functionalization, catenation, and variation of the metal oxide and organic linking units on the low-pressure hydrogen adsorption properties of metal-organic frameworks. J. Am. Chem. Soc..

[8] Murray L.J., Dinc M., Long J.R. (2009). Hydrogen storage in metal-organic frameworks. Chem. Soc. Rev..

[9] Chen B., Liang C., Yang J., Contreras D.S., Clancy Y.L., Lobkovsky E.B., Yaghi O.M., Dai S. (2006). A microporous metal-organic framework for gas-chromatographic separation of alkanes. Angew. Chem. Int. Ed..

[10] Bloch E.D., Queen W.L., Krishna R., Zadrozny J.M., Brown C.M., Long J.R. (2012). Hydrocarbon separations in a metal-organic framework with open iron(II) coordination sites. Science.

[11] Allendorf M.D., Bauer C.A., Bhakta R.K., Houk R.J.T. (2009). Luminescent metal–organic frameworks. Chem. Soc. Rev..

[12] Stassen I., Burtch N., Talin A., Falcaro P., Allendorf M., Ameloot R. (2017). An updated roadmap for the integration of metal–organic frameworks with electronic devices and chemical sensors. Chem. Soc. Rev..

[13] Sun L., Campbell M.G., Dincă M. (2016). Electrically Conductive Porous Metal–Organic Frameworks. Angew. Chem. Int. Ed..

[14] Allendorf M.D., Foster M.E., Léonard F., Stavila V., Feng P.L., Doty F.P., Leong K., Ma E.Y., Johnston S.R., Talin A.A. (2015). Guest-Induced Emergent Properties in Metal–Organic Frameworks. J. Phys. Chem. Lett..

[15] Haldar R., Heinke L., Wöll C. (2020). Advanced Photoresponsive Materials Using the Metal–Organic Framework Approach. Adv. Mater..

[16] Kreno L.E., Leong K., Farha O.K., Allendorf M., Van Duyne R.P., Hupp J.T. (2012). Metal-organic framework materials as chemical sensors. Chem. Rev..

[17] Son H.J., Jin S., Patwardhan S., Wezenberg S.J., Jeong N.C., So M., Wilmer C.E., Sarjeant A.A., Schatz G.C., Snurr R.Q. (2013). Light-harvesting and ultrafast energy migration in porphyrin-based metal-organic frameworks. J. Am. Chem. Soc..

[18] Zhang T., Lin W. (2014). Metal-organic frameworks for artificial photosynthesis and photocatalysis. Chem. Soc. Rev..

[19] Oldenburg M., Turshatov A., Busko D., Wollgarten S., Adams M., Baroni N., Welle A., Redel E., Wöll C., Richards B.S. (2016). Photon Upconversion at Crystalline Organic–Organic Heterojunctions. Adv. Mater..

[20] Thomas S., Li H., Dasari R.R., Evans A.M., Castano I., Allen T.G., Reid O.G., Rumbles G., Dichtel W.R., Gianneschi N.C. (2019). Design and synthesis of two-dimensional covalent organic frameworks with four-arm cores: Prediction of remarkable ambipolar charge-transport properties. Mater. Horiz..

[21] Clough A.J., Orchanian N.M., Skelton J.M., Neer A.J., Howard S.A., Downes C.A., Piper L.F.J., Walsh A., Melot B.C., Marinescu S.C. (2019). Room Temperature Metallic Conductivity in a Metal–Organic Framework Induced by Oxidation. J. Am. Chem. Soc..

[22] Skorupskii G., Trump B.A., Kasel T.W., Brown C.M., Hendon C.H., Dincă M. (2020). Efficient and tunable one-dimensional charge transport in layered lanthanide metal–organic frameworks. Nat. Chem..

[23] Wang M., Ballabio M., Wang M., Lin H.-H., Biswal B.P., Han X., Paasch S., Brunner E., Liu P., Chen M. (2019). Unveiling Electronic Properties in Metal–Phthalocyanine-Based Pyrazine-Linked Conjugated Two-Dimensional Covalent Organic Frameworks. J. Am. Chem. Soc..

[24] Xie L.S., Skorupskii G., Dincă M. (2020). Electrically Conductive Metal–Organic Frameworks. Chem. Rev..

[25] Tamai Y., Ohkita H., Benten H., Ito S. (2015). Exciton Diffusion in Conjugated Polymers: From Fundamental Understanding to Improvement in Photovoltaic Conversion Efficiency. J. Phys. Chem. Lett..

[26] Menke S.M., Holmes R.J. (2014). Exciton diffusion in organic photovoltaic cells. Energy Environ. Sci..

[27] Zhang Q., Zhang C., Cao L., Wang Z., An B., Lin Z., Huang R., Zhang Z., Wang C., Lin W. (2016). Förster Energy Transport in Metal-Organic Frameworks Is beyond Step-by-Step Hopping. J. Am. Chem. Soc..

[28] Adams M., Kozlowska M., Baroni N., Oldenburg M., Ma R., Busko D., Turshatov A., Emandi G., Senge M.O., Haldar R. (2019). Highly Efficient One-Dimensional Triplet Exciton Transport in a Palladium-Porphyrin-Based Surface-Anchored Metal-Organic Framework. ACS Appl. Mater. Interfaces.

[29] Kretz B., Egger D.A., Zojer E. (2015). A Toolbox for Controlling the Energetics and Localization of Electronic States in Self-Assembled Organic Monolayers. Adv. Sci..

[30] Obersteiner V., Jeindl A., Götz J., Perveaux A., Hofmann O.T., Zojer E. (2017). Electrostatic Design of 3D Covalent Organic Networks. Adv. Mater..

[31] Joshi T., Chen C., Li H., Diercks C.S., Wang G., Waller P.J., Li H., Bredas J.-L., Yaghi O.M., Crommie M.F. (2019). Local Electronic Structure of Molecular Heterojunctions in a Single-Layer 2D Covalent Organic Framework. Adv. Mater..

[32] Natan A., Kronik L., Haick H., Tung R.T. (2007). Electrostatic Properties of Ideal and Non-ideal Polar Organic Monolayers: Implications for Electronic Devices. Adv. Mater..

[33] Cahen D., Naaman R., Vager Z. (2005). The Cooperative Molecular Field Effect. Adv. Funct. Mater..

[34] Heimel G., Rissner F., Zojer E. (2010). Modeling the Electronic Properties of π-Conjugated Self-Assembled Monolayers. Adv. Mater..

[35] Monti O.L.A. (2012). Understanding Interfacial Electronic Structure and Charge Transfer: An Electrostatic Perspective. J. Phys. Chem. Lett..

[36] Zojer E., Taucher T.C., Hofmann O.T. (2019). The Impact of Dipolar Layers on the Electronic Properties of Organic/Inorganic Hybrid Interfaces. Adv. Mater. Interfaces.

[37] Rissner F., Natan A., Egger D.A., Hofmann O.T., Kronik L., Zojer E. (2012). Dimensionality effects in the electronic structure of organic semiconductors consisting of polar repeat units. Org. Electron..

[38] Goniakowski J., Finocchi F., Noguera C. (2007). Polarity of oxide surfaces and nanostructures. Rep. Prog. Phys..

[39] Dulub O., Diebold U., Kresse G. (2003). Novel Stabilization Mechanism on Polar Surfaces: ZnO(0001)-Zn. Phys. Rev. Lett..

[40] Kresse G., Dulub O., Diebold U. (2003). Competing stabilization mechanism for the polar ZnO(0001)-Zn surface. Phys. Rev. B.

[41] Ito E., Washizu Y., Hayashi N., Ishii H., Matsuie N., Tsuboi K., Ouchi Y., Harima Y., Yamashita K., Seki K. (2002). Spontaneous buildup of giant surface potential by vacuum deposition of Alq3 and its removal by visible light irradiation. J. Appl. Phys..

[42] Sze S.M., Ng K.K. (2007). Physics of Semiconductor Devices.

[43] Talin A.A., Centrone A., Ford A.C., Foster M.E., Stavila V., Haney P., Kinney R.A., Szalai V., Gabaly F.E., Yoon H.P. (2014). Tunable Electrical Conductivity in Metal-Organic Framework Thin-Film Devices. Science.

[44] Taucher T.C., Hehn I., Hofmann O.T., Zharnikov M., Zojer E. (2016). Understanding Chemical versus Electrostatic Shifts in X-ray Photoelectron Spectra of Organic Self-Assembled Monolayers. J. Phys. Chem. C.

[45] Taucher T.C., Zojer E. (2020). The Potential of X-ray Photoelectron Spectroscopy for Determining Interface Dipoles of Self-Assembled Monolayers. Appl. Sci..

[46] Mancuso J.L., Mroz A.M., Le K.N., Hendon C.H. (2020). Electronic Structure Modeling of Metal–Organic Frameworks. Chem. Rev..

[47] Blum V., Gehrke R., Hanke F., Havu P., Havu V., Ren X., Reuter K., Scheffler M. (2009). Ab initio molecular simulations with numeric atom-centered orbitals. Comput. Phys. Commun..

[48] Havu V., Blum V., Havu P., Scheffler M. (2009). Efficient O(N) integration for all-electron electronic structure calculation using numeric basis functions. J. Comput. Phys..

[49] Marek A., Blum V., Johanni R., Havu V., Lang B., Auckenthaler T., Heinecke A., Bungartz H.-J., Lederer H. (2014). The ELPA library: Scalable parallel eigenvalue solutions for electronic structure theory and computational science. J. Phys. Condens. Matter.

[50] Yu V.W., Corsetti F., García A., Huhn W.P., Jacquelin M., Jia W., Lange B., Lin L., Lu J., Mi W. (2018). ELSI: A unified software interface for Kohn–Sham electronic structure solvers. Comput. Phys. Commun..

[51] Neugebauer J., Scheffler M. (1992). Adsorbate-substrate and adsorbate-adsorbate interactions of Na and K adlayers on Al(111). Phys. Rev. B.

[52] Freysoldt C., Eggert P., Rinke P., Schindlmayr A., Scheffler M. (2008). Screening in two dimensions: $GW$ calculations for surfaces and thin films using the repeated-slab approach. Phys. Rev. B.

[53] Perdew J.P., Burke K., Ernzerhof M. (1996). Generalized Gradient Approximation Made Simple. Phys. Rev. Lett..

[54] Perdew J.P., Burke K., Ernzerhof M. (1997). Generalized Gradient Approximation Made Simple [Phys. Rev. Lett. 77, 3865 (1996)]. Phys. Rev. Lett..

[55] Tkatchenko A., Scheffler M. (2009). Accurate Molecular Van Der Waals Interactions from Ground-State Electron Density and Free-Atom Reference Data. Phys. Rev. Lett..

[56] van Lenthe E., Baerends E.J., Snijders J.G. (1993). Relativistic regular two‐component Hamiltonians. J. Chem. Phys..

[57] Bagus P.S., Ilton E.S., Nelin C.J. (2013). The interpretation of XPS spectra: Insights into materials properties. Surf. Sci. Rep..

[58] Perdew J.P., Norman M.R. (1982). Electron removal energies in Kohn-Sham density-functional theory. Phys. Rev. B.

[59] Stowasser R., Hoffmann R. (1999). What Do the Kohn−Sham Orbitals and Eigenvalues Mean?. J. Am. Chem. Soc..

[60] Chong D.P., Gritsenko O.V., Baerends E.J. (2002). Interpretation of the Kohn–Sham orbital energies as approximate vertical ionization potentials. J. Chem. Phys..

[61] Heyd J., Scuseria G.E., Ernzerhof M. (2003). Hybrid functionals based on a screened Coulomb potential. J. Chem. Phys..

[62] Krukau A.V., Vydrov O.A., Izmaylov A.F., Scuseria G.E. (2006). Influence of the exchange screening parameter on the performance of screened hybrid functionals. J. Chem. Phys..

[63] Kokalj A. (1999). XCrySDen—a new program for displaying crystalline structures and electron densities. J. Mol. Graph. Model..

[64] Stukowski A. (2010). Visualization and analysis of atomistic simulation data with OVITO–the Open Visualization Tool. Model. Simul. Mater. Sci. Eng..

[65] Shekhah O., Wang H., Kowarik S., Schreiber F., Paulus M., Tolan M., Sternemann C., Evers F., Zacher D., Fischer R.A. (2007). Step-by-Step Route for the Synthesis of Metal−Organic Frameworks. J. Am. Chem. Soc..

[66] Zacher D., Shekhah O., Wöll C., Fischer R.A. (2009). Thin films of metal–organic frameworks. Chem. Soc. Rev..

[67] Makiura R., Motoyama S., Umemura Y., Yamanaka H., Sakata O., Kitagawa H. (2010). Surface nano-architecture of a metal–organic framework. Nat. Mater..

[68] Liu J., Wöll C. (2017). Surface-supported metal–organic framework thin films: Fabrication methods, applications, and challenges. Chem. Soc. Rev..

[69] Yu X.-J., Xian Y.-M., Wang C., Mao H.-L., Kind M., Abu-Husein T., Chen Z., Zhu S.-B., Ren B., Terfort A. (2019). Liquid-Phase Epitaxial Growth of Highly Oriented and Multivariate Surface-Attached Metal–Organic Frameworks. J. Am. Chem. Soc..

[70] Wang Z., Liu J., Lukose B., Gu Z., Weidler P.G., Gliemann H., Heine T., Wöll C. (2014). Nanoporous Designer Solids with Huge Lattice Constant Gradients: Multiheteroepitaxy of Metal–Organic Frameworks. Nano Lett..

[71] Gierschner J., Cornil J., Egelhaaf H.-J. (2007). Optical Bandgaps of π-Conjugated Organic Materials at the Polymer Limit: Experiment and Theory. Adv. Mater..

[72] Hehn I., Schuster S., Wächter T., Abu-Husein T., Terfort A., Zharnikov M., Zojer E. (2016). Employing X-ray Photoelectron Spectroscopy for Determining Layer Homogeneity in Mixed Polar Self-Assembled Monolayers. J. Phys. Chem. Lett..

[73] Methfessel M., Fiorentini V., Oppo S. (2000). Connection between charge transfer and alloying core-level shifts based on density-functional calculations. Phys. Rev. B.

[74] Morikawa Y., Hayashi T., Liew C.C., Nozoye H. (2002). First-principles theoretical study of alkylthiolate adsorption on Au(111). Surf. Sci..

[75] Heimel G., Romaner L., Brédas J.-L., Zojer E. (2006). Organic/metal interfaces in self-assembled monolayers of conjugated thiols: A first-principles benchmark study. Surf. Sci..

[76] Bellafont N.P., Illas F., Bagus P.S. (2015). Validation of Koopmans’ theorem for density functional theory binding energies. Phys. Chem. Chem. Phys..

[77] El-Sayed A., Borghetti P., Goiri E., Rogero C., Floreano L., Lovat G., Mowbray D.J., Cabellos J.L., Wakayama Y., Rubio A. (2013). Understanding Energy-Level Alignment in Donor–Acceptor/Metal Interfaces from Core-Level Shifts. ACS Nano.

[78] Ulman A. (1996). Formation and Structure of Self-Assembled Monolayers. Chem. Rev..

[79] Schreiber F. (2000). Structure and growth of self-assembling monolayers. Prog. Surf. Sci..

[80] Love J.C., Estroff L.A., Kriebel J.K., Nuzzo R.G., Whitesides G.M. (2005). Self-Assembled Monolayers of Thiolates on Metals as a Form of Nanotechnology. Chem. Rev..

[81] Vericat C., Vela M.E., Benitez G., Carro P., Salvarezza R.C. (2010). Self-assembled monolayers of thiols and dithiols on gold: New challenges for a well-known system. Chem. Soc. Rev..

[82] Topping J. (1927). On the Mutual Potential Energy of a Plane Network of Doublets. Proc. R. Soc. A Math. Phys. Eng. Sci..

[83] Marder S.R., Kippelen B., Jen A.K.-Y., Peyghambarian N. (1997). Design and synthesis of chromophores and polymers for electro-optic and photorefractive applications. Nature.

[84] Gu C., Zhang H., You P., Zhang Q., Luo G., Shen Q., Wang Z., Hu J. (2019). Giant and Multistage Nonlinear Optical Response in Porphyrin-Based Surface-Supported Metal–Organic Framework Nanofilms. Nano Lett..

