# Supplementary material for: Electrostatic Design of Polar Metal–Organic Framework Thin Films
Source: Nanomaterials (Basel). 2020 Dec 3;10(12):2420. doi: 10.3390/nano10122420 (PMC7761790; doi:10.3390/nano10122420)
Supplement: Supplementary file 1 [file nanomaterials-10-02420-s001.pdf]

nanomaterials

Supplementary Materials

Electrostatic Design of Polar Metal–Organic Framework Thin Films

Giulia Nascimbeni [1], Christof Wöll [2] and Egbert Zojer [1,*]

[1] Institute of Solid State Physics, NAWI Graz, Graz University of Technology, Petersgasse 16, 8010 Graz, Austria; nascimbenigiulia@gmail.com
[2] Institute of Functional Interfaces (IFG), Karlsruhe Institute of Technology (KIT), Hermann-von-Helmholtz Platz-1, 76344 Eggenstein-Leopoldshafen, Germany; christof.woell@kit.edu
* Correspondence: Egbert.zojer@tugraz.at

1. Examples for Studied Unit Cells

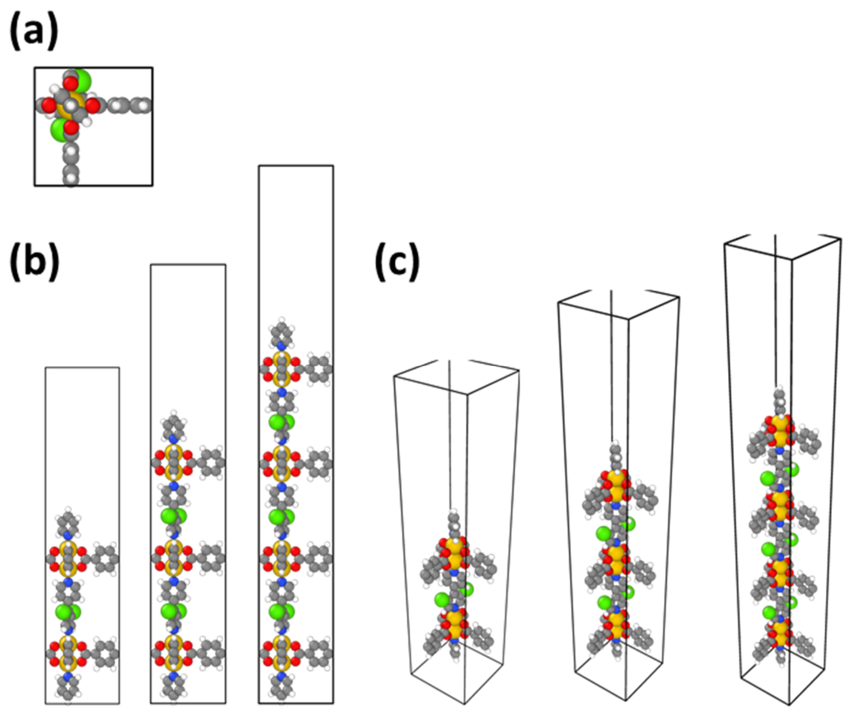

Figure S1. Top (**a**), side (**b**), and diagonal (**c**) views of the unit cells used in the mono- bi- and trilayer Simulations (the top views are identical for all three systems) C: grey spheres; O: red spheres; H: small white spheres; F: green spheres, Zn: yellow spheres.

2. Methodological Aspects

2.1. Details on the Used Basis Sets

The basis functions employed in the FHI-aims simulations have the format

$$\Phi(r) = \frac{u(r)}{r} \times Y_{lm}(\Theta, \Phi)$$

In spherical coordinates (r, Θ, Φ) relative to a given atomic center. FHI-aims provides for every atomic species a preconstructed *species_defaults* file. The used light basis sets were not further adjusted

apart from adding tier 2 basis functions (see following table), because they afforded the required accuracy and efficiency.

Table S1. Basis functions that have been used for all calculations performed with FHI-aims [1]. The abbreviations read as follows : X(nl, z), where X describes the type of basis function where H stands for hydrogen-like functions and ionic for a free-ion like radial function. The parameter n stands for the main/radial quantum number, l denotes the angular momentum quantum number (s, p, d, f, …), and z denotes an effective nuclear charge, which scales the radial function in the defining Coulomb potential for the hydrogen-like function. In the case of free-ion like radial functions, z specifies the onset radius of the confining potential. If auto is specified instead of a numerical value, the default onset is used.

	**H**	**C**	**N**	**O**	**F**	**Zn**
Minimal	valence (1s, 1.0)	valence (2s, 2.0)<br>Valence (2p, 2.0)	valence (2s, 2.0)<br>valence (2p, 3.0)	valence (2s, 2.0)<br>valence (2p, 4.0)	valence (2s, 2.0)<br>valence (2p, 5.0)	valence (4s, 2.0)<br>valence (3p, 6.0)<br>valence (3d, 10.0)
First tier	H(2s, 2.1)<br>H(2p, 3.5)	H(2p, 1.7)<br>H(3d, 6)<br>H(2s, 4.9)	H(2p, 1.8)<br>H(3d, 6.8)<br>H(3s, 5.8)	H(2p, 1.8)<br>H(3d, 7.6)<br>H(3s, 6.4)	H(2p, 1.7)<br>H(3d, 7.4)<br>H(3s, 6.8)	H(2p, 1.7)<br>H(3s, 2.9)<br>H(4p, 5.4)<br>H(4f, 7.8)<br>H(3d, 4.5)
Second tier	H(1s, 0.85)<br>H(2p, 3.7)<br>H(2s, 1.2)<br>H(3d, 7.0)	H(4f, 9.8)<br>H(3p, 5.2)<br>H(3s, 4.3)<br>H(5g, 14.4)<br>H(3d, 6.2)	H(4f, 10.8)<br>H(3p, 5.8)<br>H (1s, 0.8)<br>H(5g, 16)<br>H(3d, 4.9)	H(4f, 11.6)<br>H(3p, 6.2)<br>H (3d, 5.6)<br>H(5g, 17.5)<br>H(1s, 0.75)	H(4f, 11.2)<br>ionic (2p, auto)<br>H (1s, 0.75)<br>H(4d, 8.8)<br>H(5g, 16.8)	H(5g, 10.8)<br>H(H2p, 2.4)<br>H(3s, 6.2)<br>H(3d, 3)

[1] As described in the FHI-aims manual, version January 23, 2017.

2.2. Impact of the Van Der Waals Correction:

We tested the impact of the van der Waals corrections in the geometry optimizations and total energy calculations for the m2F-BP and o2F-BP-derived clusters and found that including them had no impact on the calculated dipoles (with changes for the up-, down- and molecular systems by < 0.02 Debye), as shown in Table S2. This strongly suggests that including van der Waals corrections has no impact on any of the reported electronic properties of the MOFs. Conversely, the binding energies, of course, increased significantly when including van der Waals corrections. The impact on the bonding asymmetry of up- vs. down configurations was, however, again rather minor. −66 (467) vs. −71 (488) meV for m2F-BP (o2F-BP) with and without van der Waals corrections (see Table S2).

Table S2. Comparison of dipole moments (μ_{mol}, μ_{down}, μ_{up}), binding energies ($E_{B,down}$, $E_{b,up}$) and binding energy asymmetries (DE_b) of the m2F-BP and o2F-BP-derived clusters in their up- and down configurations and for the isolated molecules depending on whether or not a Tkatchenko-Scheffler type van der Waals correction [2] had been used in the geometry optimization process. Data plotted in red are also contained in the main manuscript. Data plotted in italics are for the o2F-BP apical linker.

	**m2F-BP vdW**	**o2F-BP vdW**	**m2F-BP no-vdW**	**o2F-BP no-vdW**
μ_{mol} (D)	−0.76	−1.86	−0.77	−1.86
μ_{down} (D)	−3.57	−4.27	−3.59	−4.26
μ_{up} (D)	−1.40	−0.63	−1.40	−0.64
$E_{b,down}$ (meV)	995	1057	763	830
$E_{b,up}$ (meV)	1061	590	833	341
ΔE_b (meV)	**−66**	**467**	**−71**	**488**

2.3. Impact of Basis-Set Superposition Error:

To test the role of the basis set for the calculation of binding energies (and especially for their asymmetries), we tested an extended (tier 3) basis set for the m2F-BP and o2F-BP systems and also performed counterpoise correction. As shown in Table S3, increasing the basis set size resulted in variations of the binding energies by a few meV (<< 1%). Most importantly, the change in the asymmetry of the binding energies for up and down configurations was only ≤ 1 meV. The same is observed for calculations employing a counterpoise correction (with somewhat larger changes for the absolute binding energies). Here, for technical reasons binding energies and molecular dipoles have been calculated for the geometries of all constituents fixed to the geometries they adopt in the cluster (i.e., geometry optimizations for constituents with the counterpoise correction switched on are not really sensible). Therefore, for assessing the impact of the counterpoise correction the two "tier2/fixed" columns for regular calculations at fixed geometries without counterpoise correction) need to be compared to the results of the calculations with counterpoise correction (last two columns of Table S3).

Table S3. Comparison of dipole moments (μ_{mol}, μ_{down}, μ_{up}), binding energies ($E_{B,down}$, $E_{b,up}$) and binding energy asymmetries (ΔE_b) of the m2F-BP and o2F-BP-derived clusters in their up- and down configurations and for the isolated molecules as a function of the used basis sets. tier2/opt. are values obtained for simulations with the tier 2 basis set specified above in which not only the cluster geometries, but also the geometries of its isolated constituents (nodes and linkers) have been optimized; for tier3/opt. the same procedure has been applied but now including the full tier3 basis sets (again with light settings). tier2 fixed refers again to tier 2 calculations, but for calculating binding energies and molecular dipoles the linker molecule and node have been fixed to the geometries they adopt in the cluster. Finally, CP refers to values obtained with tier 2 calculations of linker and note (based on the cluster geometries), in which for the total energy calculations a counterpoise correction has been performed. Data plotted in red are also contained in the main manuscript. Data plotted in italics are for the o2F-BP apical linker.

	**m2F-BP tier2/opt.**	**o2F-BP tier2/opt.**	**m2F-BP tier3/opt.**	**o2F-BP tier3/opt.**	**m2F-BP tier2/fixed**	**o2F-BP tier2/fixed**	**m2F-BP CP**	**o2F-BP CP**
μ_{mol} (D)	−0.76	−1.86	−0.76	−1.84	−0.81	−1.91	-	-
μ_{down} (D)	−3.57	−4.27	−3.57	−4.24	−3.57	−4.27	-	-
μ_{up} (D)	−1.40	−0.63	−1.43	−0.64	−1.40	−0.63	-	-
$E_{b,down}$ (meV)	995	1057	990	1052	1277	1367	1264	1353
$E_{b,up}$ (meV)	1061	590	1056	586	1389	885	1375	871
ΔE_b (meV)	**−66**	**467**	**−66**	**466**	**111**	**−482**	**111**	**−482**

3. Plane-Averaged Electrostatic Energy

In addition to plotting the positional dependence of the electrostatic energy in an isodensity plot (as in Figure 3 of the main manuscript), one can also plot the electrostatic energy averaged over the xy-plane. This is shown for the 7-layer system (n = 7) in Figure S2 and for the model of an analogue to a pin junction in Figure S3.

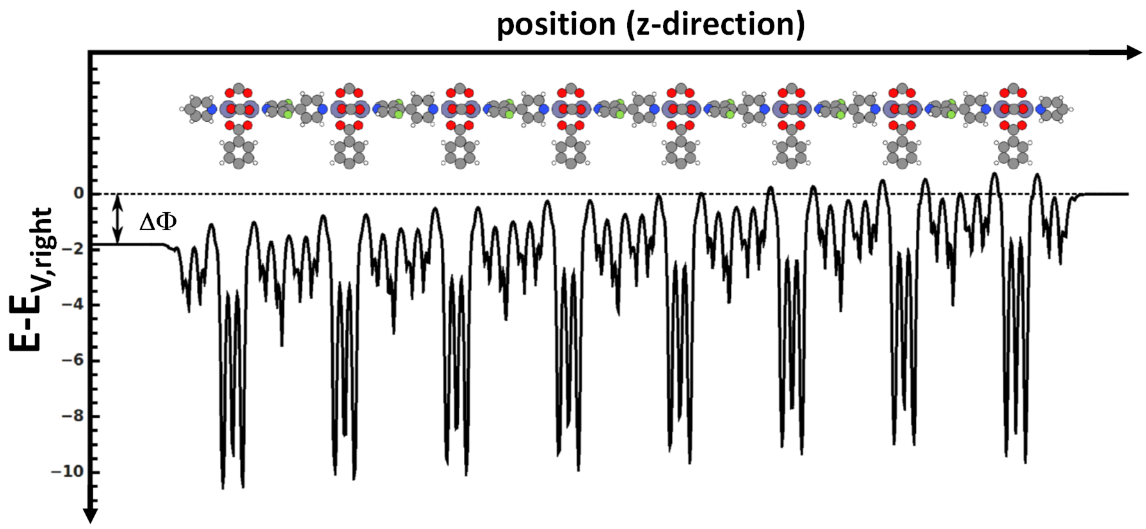

Figure S2. PBE calculated, electrostatic energy averaged over the xy-plane and plotted as a function of the z-coordinate for the 7-layer system. The energy is aligned to the right vacuum level. The energy difference ΔΦ between the two sides of the sample is indicated by the arrow. The structure of the unit cell is shown as an inset denoting also the positions of the minima in energy.

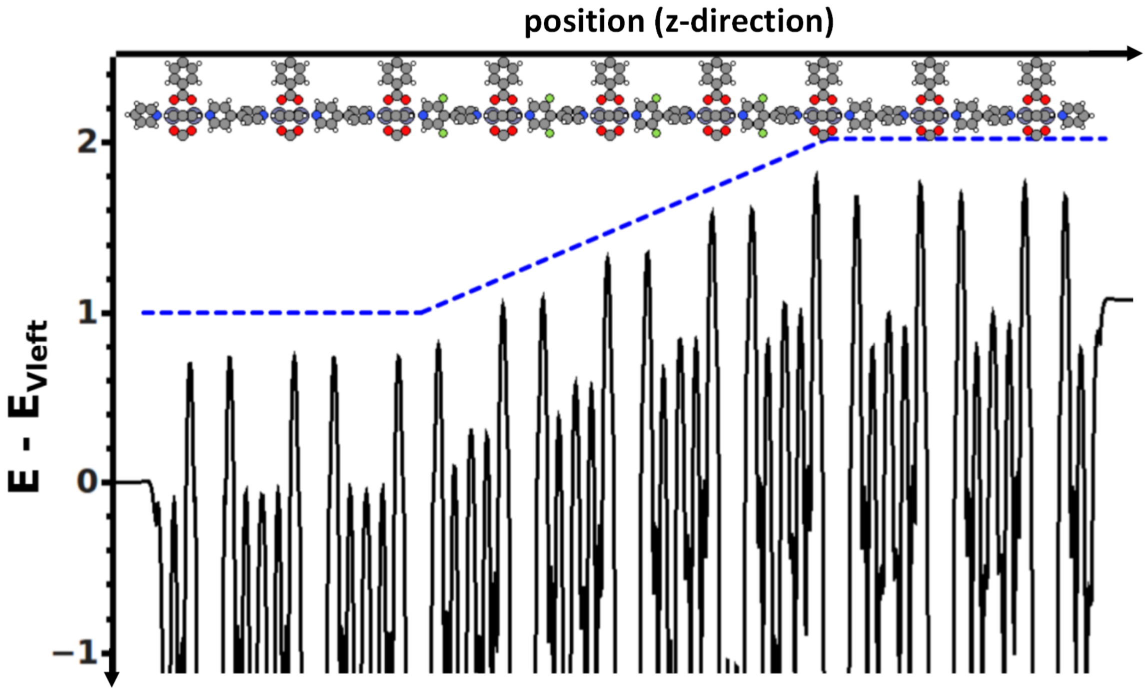

Figure S3. PBE calculated, electrostatic energy averaged over the xy-plane and plotted as a function of the z-coordinate for a system containing two layers of apolar 4,4′-bipyridine linkers, followed by four layers of polar 3,5-difluoro-4,4′-bipyridine linkers and finally by another two 4,4′-bipyridine linkers. The structure of the unit cell is shown as an inset denoting also the positions of the minima in energy.

4. Density of States in the PBE Calculations for the 7-Layer System Plotted over a Wider Energy Range

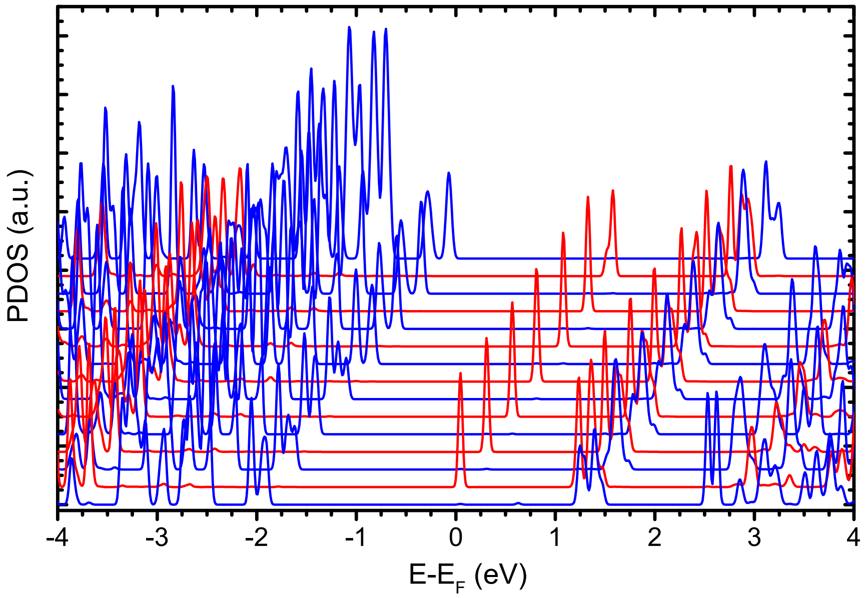

Figure S4. Density of states for the 7-layer system projected onto successive BDC-linked Zn-paddlewheel layers (blue) and m2F-BP layers (red). In analogy to Figure 4a in the main manuscript, but plotted over a wider energy range.

5. Structures of All "Monomer" Systems

To calculate the bonding energies of the polar apical linkers and their asymmetries, we simulated various linkers bonded to a single, saturated Zn-paddlewheel. The optimized structures of all considered systems are shown in Figure S5.

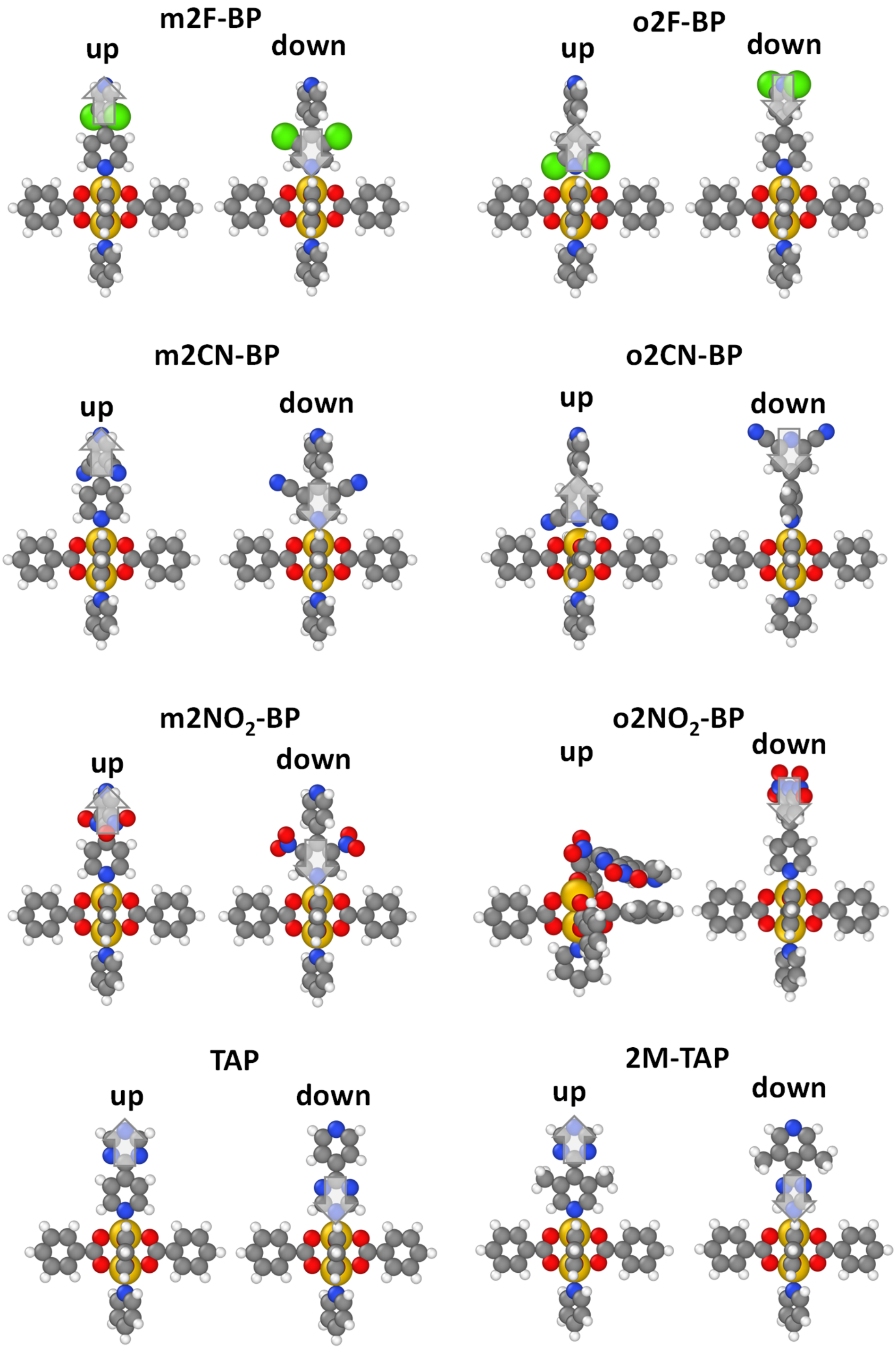

Figure S5. Structures of the up and down configurations of saturated Zn-paddlewheel monomers bonded to polar apical linkers (yellow: C; cyan: H; dark blue: N; red: O; violet: Zn): m2F-BP: 3,5-difluoro-4,4′-bipyridine; o2F-BP: 2,6-difluoro-4,4′-bipyridine; m2CN-BP: 3,5-dicyano-4,4′-bipyridine; o2CN-BP: 2,6-dicyano-4,4′-bipyridine; m2NO$_2$-BP: 3,5-dinitro-4,4′-bipyridine; o2NO$_2$-BP: 2,6-dinitro-4,4′-bipyridine; TAP: 4-s-triacinylpyridine; 2M-TAP: 3,5-dimetil-4-s- triacinylpyridine linker. C: yellow spheres; O: red spheres; H: small cyan spheres; F: green spheres, Zn: large, purple spheres.

6. Dipole per Unit Cell per Polar Layer

Assuming that molecular dipoles and bonding dipoles of the monomeric units from Figure S5 can be simply superimposed, one can calculate a rough estimate of the dipole per unit cell per layer of polar apical linkers.

$$\Delta\mu = \mu_{down} - \mu_{up} - \mu_{mol}$$

This can be rationalized by the following considerations: The dipole of μ_{down} consists of the dipole of the Zn-N bond for the "down" oriented linker plus the molecular dipole; the dipole of μ_{up} consists of the dipole of the Zn-N bond for the "up" oriented linker and the rotated molecules (i.e., plus $-\mu_{mol}$). When subtracting μ_{up} from μ_{down} that corresponds to rotating the structure of the up configuration, which is exactly what one needs to correctly account for the bond dipoles. The molecular dipole is, however counted twice (as $-(-\mu_{mol}) = +\mu_{mol}$ from the rotated monomer "up"-configuration. To account for that, μ_{mol} has to be subtracted to get $\Delta\mu$.

7. Comparison of the Electronic Properties of the m2F-BP and o2F-BP Monolayers Infinitely Extended in the Directions Perpendicular to the Dipole Direction

Table S4. Comparison between the global band gap, $E_{G,global}$, and the work function change $\Delta\Phi$ of the first layer for the two different difluorobipyridine linkers. For the definition of the band gap and the work function change see main text.

	$E_{G,global}$ (eV)	$\Delta\Phi$ (eV)
3,5-difluoro-4,4'-bipyridine (m2F-BP)	1.60	0.27
2,6-difluoro-4,4'-bipyridine (o2F-BP)	1.39	−0.29

8. Check for an Activation Energy in the Bond-Formation Process

In order to more clearly assess the role of kinetics in the bond-formation process, we also checked, whether an activation energy would have to be overcome during bond formation. To that aim, the energy of the "up" m2F-BP-monomer (Figure S5, top left structure) was evaluated as a function of the distance between the apical linker and the saturated Zn-paddlewheel, starting from the equilibrium distance. The results shown in Figure S6 indicate that there is no activation energy for the bonding process. Qualitatively the same results were obtained for the "down" system.

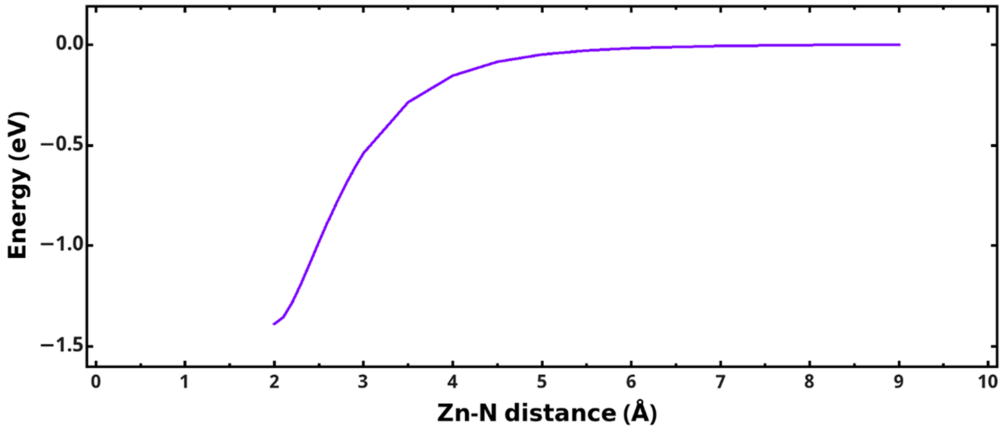

Figure S6. PBE-calculated total energy of the "up" m2F-BP-monomer as a function of the distance between the Zn-atom of the saturated paddlewheel and the N atom in the apical linker. The curve starts at the equilibrium distance of the bond at 2.08 Å. The energies are given relative to the value at infinite distance and they have been obtained via single-point calculations without optimizing the structures at every distance.

9. Dipole-Dipole Interactions within Monolayers

To check whether the interactions between neighboring dipole units play a role, slab calculations for the three systems depicted in Figure S7 were performed. In the "up" and "down" configurations, the dipole units are parallel. Instead, in the "checkerboard" system the orientation of the dipoles is

alternate. Note that the systems studied here consist of only one layer of saturated Zn-paddlewheels connected by BDC linkers to which a monolayer of apical linkers has been bonded. I.e., the top-layer of BDC-linked paddlewheels is missing, as otherwise no insights on the bonding asymmetry could be obtained. The bonding energies are compared in Table S5. Contrary to what one might expect based on dipole-dipole attraction/repulsion arguments, the "checkerboard" structure is not the most stable one. In fact, the bonding energy of that structure is essentially half way between the "up" and "down" configurations. This shows that dipole-dipole interactions play an only negligible role, which can be attributed to the comparably small encountered dipole moments and especially to the large inter-dipole distances of the highly porous systems (inter-dipole distances of about 11Å). This assessment is also backed by simple electrostatic arguments (see main manuscript).

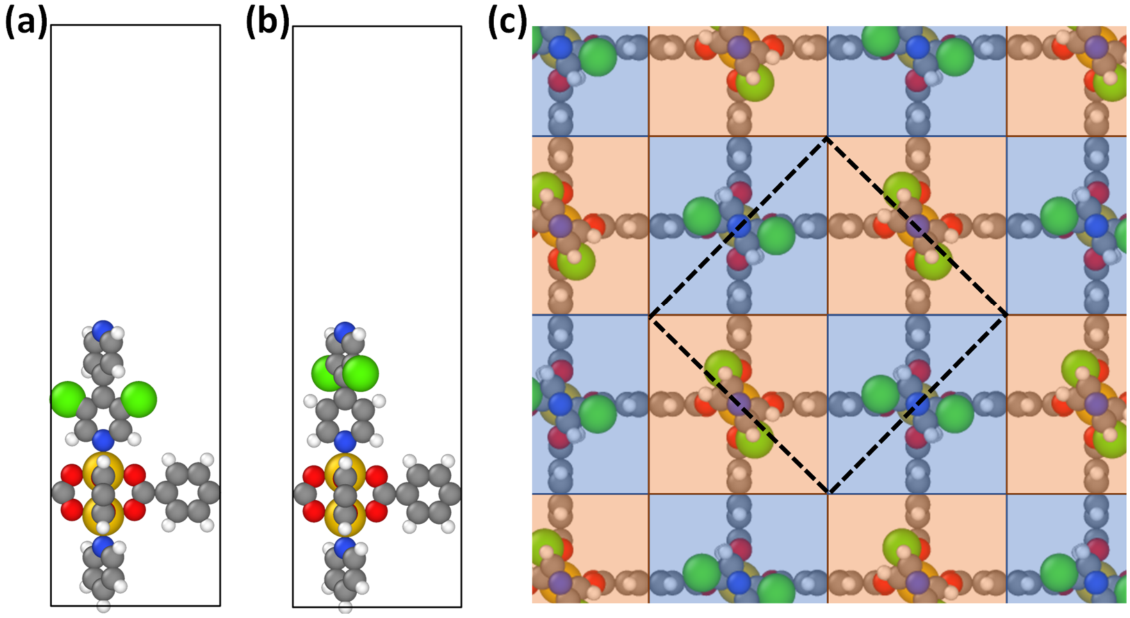

Figure S7. Systems used for slab calculations, to investigate the interactions between adjacent dipole units. In panels (**a**) and (**b**) side-views of the "down" (left) and "up" (right) systems are depicted. Panel (**c**) shows a top-view of the "checkerboard system", where different shadings indicate dipole-up and down orientations and the dashed black square shows the unit cell (containing on m2F-BP and one o2F-BP molecule). C atoms are depicted in grey, H atoms in white, O atoms in red, N in blue, Zn in yellow, and F in green.

Table S5. Comparison of the bonding energies, E_b, for the "up", "down" and "checkerboard" slab systems infinitely extended in c- and y-directions.

	E_b (meV)	ΔE_b (meV)
up	1108	-
down	1048	−60 meV
checkerboard	1082	-

10. Interaction between Polar Molecules in Neighboring Layers

To quantify the interaction between polar molecules in neighboring layers, we performed simulations on dimer clusters as well as on slabs containing two periodic layers of apical linkers. In contrast to the n = 2 situation in Figure S1b, only two Zn-paddlewheels (or BDC-linked layers of Zn-paddlewheels) are considered in analogy to the situation for the monolayers shown in Figure S7. This is necessary to be able to calculate bonding asymmetries. Bonding energies are then calculated here for the removal of the topmost m2F-BP molecule or monolayer (where for the latter the energy is given again per apical linker molecule).

An analysis of the bonding energies Table S6 reveals that again, there is virtually no interaction between m2F-BP in neighboring layers, as the differences in bonding energies for open as well as for periodic boundary conditions are essentially identical to the monomer value reported in Table 1 of the main manuscript (−66 meV). This highlights that also here the asymmetry in the difference in bonding energies primarily originates from the orientation of the topmost m2F-BP relative to the Zn-paddlewheel it is bonded to.

Table S6. Comparison of the bonding energies, E_b, of dimer clusters (containing two polar molecules and two saturated Zn-paddlewheels stacked on top of each other with either identical or opposite orientations of the dipoles) with the results of equivalent periodic systems infinitely extended in x- and y-direction. The reported values describe the energy cost of removing of the topmost m2F-BP molecule/monolayer.

System	Dipole Orientation	E_b (meV)	ΔE_b (meV)
cluster	identical	1022	-
	opposite	1090	−68
periodic slab	identical	1082	-
	opposite	1141	−59

References

1. V. Blum, R. Gehrke, F. Hanke, P. Havu, V. Havu, X. Ren, K. Reuter, M. Scheffler, *Computer Physics Communications* **2009**, *180*, 2175.
2. A. Tkatchenko, M. Scheffler, *Physical Review Letters* **2009**, *102*, DOI 10.1103/PhysRevLett.102.073005.

© 2020 by the authors. Licensee MDPI, Basel, Switzerland. This article is an open access article distributed under the terms and conditions of the Creative Commons Attribution (CC BY) license (http://creativecommons.org/licenses/by/4.0/).
